# Multiple liver abscesses and bacteremia caused by *Streptococcus constellatus* infection: a case report

**DOI:** 10.1002/ccr3.774

**Published:** 2016-12-20

**Authors:** Nobuhiro Akuzawa, Takashi Hatori, Yonosuke Kitahara, Masahiko Kurabayashi

**Affiliations:** ^1^ Department of General MedicineNational Hospital Organization Shibukawa Medical Center383 ShiroiShibukawaGunma377‐0280Japan; ^2^ Department of Internal MedicineJapan Community Health Care Organization Gunma Chuo Hospital1‐7‐13 Koun‐choMaebashiGunma371‐0025Japan; ^3^ Department of Medicine and Biological ScienceGunma University Graduate School of Medicine3‐39‐22 Showa‐machiMaebashiGunma371‐8511Japan

**Keywords:** Abdominal ultrasonography, computed tomography, diverticulitis, liver abscess, *Streptococcus milleri* group

## Abstract

The *Streptococcus milleri* group (SMG) comprises commensal mucosal bacteria. Pyogenic liver abscesses due to hematogenous SMG infection are rare but can be observed even in healthy patients. In such cases, physicians should consider the existence of primary lesions that allow penetration of the SMG.

## Introduction

The *Streptococcus anginosus* group, also known as the *S. milleri* group (SMG), comprises *S. constellatus*,* S. intermedius*, and *S. anginosus*. These bacteria are primarily commensals of the mucosa, having been widely detected in the mouth and upper respiratory, gastrointestinal, and urogenital tracts 1, 2, 3, 4. Streptococcus milleri strains show various hemolytic, serological, and physiological characteristics that are common to viridans streptococci 1, 2, 3, 4. The pathogenic potential of the SMG has been disregarded because of the commensal nature of these microorganisms; however, streptococci belonging to this group have been increasingly reported as relevant pathogens in abscesses and blood cultures 4. Specifically, lung abscesses in adults, intracranial infection resulting from rhinosinusitis in pediatric patients, sepsis associated with surgical infection, and bacterial endocarditis due to SMG infection have been reported 5, 6, 7, 8. In addition, recent studies have suggested that the SMG has multiple virulence factors, as do other streptococcal species 4.

We herein report a rare case of an adult presenting with multiple liver abscesses, intraportal thrombosis, and bacteremia due to *S. constellatus* with no history of any surgical procedure. Results of investigations performed during his hospital stay suggested that diverticulitis of the sigmoid colon and subsequent hematogenous spread of *S. constellatus* may have led to formation of the liver abscesses and bacteremia.

## Case Report

A 69‐year‐old Japanese man was admitted to our hospital because of a 2‐day history of a fever of unknown origin. He had taken losartan (25 mg/day) and benidipine (4 mg/day) for hypertension for the preceding 10 years, but he had no other relevant medical or family history. He had been immunocompetent and had no history of diabetes mellitus. He was a nonsmoker and did not consume alcohol. One week before admission, he had presented with a slight fever (37.4°C) and left lower abdominal pain that had completely resolved within a few days.

**Figure 1:**
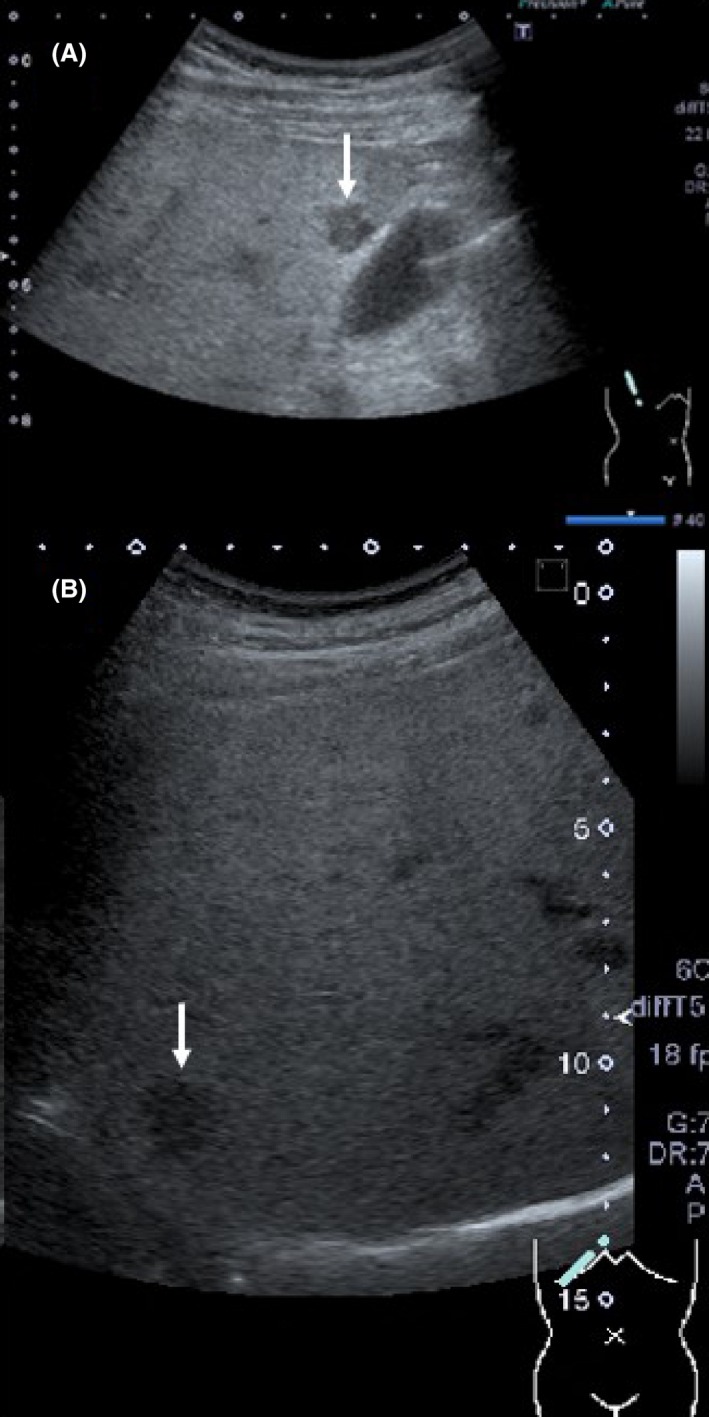
Ultrasonographic images of the liver on admission. (A) An irregularly shaped, 1‐cm‐diameter, low‐echoic focal lesion was observed in S5 adjacent to the gallbladder (white arrow). At least two other lesions showing similar characteristics and size were detected in S5; these lesions were not detected on contrast‐enhanced abdominal computed tomography. (B) A larger (2‐cm‐diameter) low‐echoic lesion was also observed in S6 (white arrow). This was the only lesion detected on contrast‐enhanced computed tomography of the abdomen.

**Figure 2:**
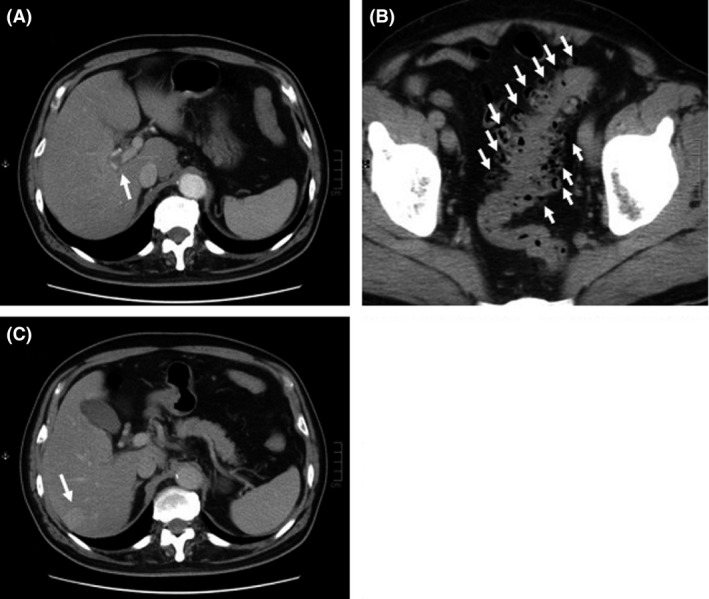
Contrast‐enhanced computed tomography images of the abdomen on admission. (A) Intravascular thrombosis was observed in the right portal branch (white arrow). (B) Multiple diverticulae were also observed in the sigmoid colon (white arrows). (C) An approximately 2‐cm‐diameter lesion was observed in S7; this was slightly enhanced in the equilibrium phase.

On admission, his height was 168 cm, weight 75.8 kg, body temperature 38.8°C, and blood pressure 128/80 mmHg. His heart rate was 92 beats per min and regular. Physical examination revealed no significant abnormalities. Both his arterial blood oxygen saturation (98%) and partial oxygen pressure (94.3 Torr) were normal. Chest and abdominal X‐ray films and an electrocardiogram were normal. Laboratory tests showed a high white blood cell count of 18.2 × 10^9^ cells/L (normal: 3.5–9.0 × 10^9^), alanine transaminase concentration of 58 U/L (normal: 5–40 U/L), alkaline phosphatase concentration of 559 U/L (normal: 115–359 U/L), *γ*‐glutamyltransferase concentration of 274 U/L (normal: <55 U/L), C‐reactive protein concentration of 20.98 mg/dL (normal: <0.3 mg/dL), plasma fibrinogen concentration of 766 mg/dL (normal: 150–350 mg/dL), prothrombin time–international normalized ratio of 1.33 (normal: 0.85–1.15), plasma fibrin/fibrinogen degradation products concentration of 6.1 *μ*g/mL (normal: <5.0 *μ*g/mL), and plasma D‐dimer concentration of 2.1 *μ*g/mL (normal: <1.0 *μ*g/mL); all parameters were above the normal range. The patient's hemoglobin A1c concentration was 5.5% (normal: <5.6%). Urinary findings were normal. Ultrasonography of the abdomen (Abd‐US) was therefore performed; this showed several low‐echoic and irregularly shaped focal hepatic lesions without surrounding capsules distributed in S5 and S6. These lesions did not show bloodstream signals on color Doppler ultrasonography, suggesting that they were liver abscesses rather than liver tumors. Most of the lesions in S5 were approximately 10 mm in diameter (Fig. 1A); however, the largest lesion (22 mm in diameter) was observed in S6 (Fig. 1B). Abd‐US also showed a mildly enlarged right hepatic lobe, mild fatty liver, and bilateral renal cysts; no other significant abnormalities were observed. Notably, contrast‐enhanced dynamic computed tomography (CT) of the abdomen (Abd‐CT) showed intravascular thrombosis in the right branch of the portal vein (Fig. 2A), multiple diverticulae, and thickening of the wall of the sigmoid colon (Fig. 2B). Although there was no obvious evidence of active inflammation, these findings raised the possibility of diverticulitis as the source of infection. In addition, a slightly and homogenously enhanced lesion in the equilibrium phase without rapid fill‐in, early washout, or a prolonged enhancement pattern was observed in the liver (S6) (Fig. 2C); however, the smaller lesions in S5 that had been detected on Abd‐US could not be seen on the Abd‐CT images. Intravenous administration of meropenem (2.0 g/day) and unfractionated heparin (15,000 units/day) was immediately begun. We strongly recommended that the patient undergo abdominal magnetic resonance imaging for detailed evaluation of the liver lesions, but he refused because of severe claustrophobia. Instead, ultrasound‐guided percutaneous liver biopsy of the S6 lesion was performed, and the obtained specimen was sent out for pathological and bacteriological examination.

On day 2, the patient's temperature remained high, and gram‐positive cocci were detected in blood culture samples obtained soon after admission; gram‐positive cocci were simultaneously detected from a liver biopsy specimen, and these were identified as *S. constellatus* on day 3. Also on day 3, echocardiography and a dental consultation were performed to check for evidence of bacterial endocarditis and untreated tooth decay or periodontal disease; these examinations showed no abnormalities. Antimicrobial susceptibility testing of the detected *S. constellatus* from both the blood culture samples and liver tissue (reported on day 5) showed the same susceptibility and no resistance to the following antibiotics: penicillin, cephems, carbapenems, fluoroquinolones, tetracyclines, or macrolides. Histopathological examination of the liver tissue reported on day 5 also showed neutrophil infiltration and pus formation (data not shown). Therefore, ampicillin (8 g/day) and gentamycin (160 mg/day) were substituted for meropenem on day 6, after which the patient's fever gradually resolved. Oral warfarin (3.0 mg/day) was commenced on day 10. Although the mass lesions in S5 and S6 of the liver persisted and were unchanged in size according to Abd‐US, the intensity of their inner echoes increased slightly on day 14. Intravenous heparin administration was terminated on day 15. Both the white blood cell count and C‐reactive protein concentration were normal on day 17 (4500/mm^3^ and 0.18 mg/dL, respectively). Colonoscopy on day 21 revealed multiple diverticulae and conspicuous wall stiffness adjacent to the sigmoid colon diverticulae, suggesting the possibility of recurrent diverticulitis. On day 22, the patient's laboratory data, including the liver enzyme concentrations, had completely returned to normal and the intravenous antibiotic treatment was stopped. The patient was discharged on day 23. After discharge, he underwent 2 weeks of oral ampicillin treatment and ongoing warfarin therapy. One month later, his laboratory data remained normal. Three months after discharge, the hepatic mass lesion and intraportal thrombosis had resolved on enhanced Abd‐CT. Abd‐US likewise revealed disappearance of the previously visible multiple low‐echoic lesions. After 4 months, the warfarin administration was terminated and he was lost to follow‐up.

## Discussion

Liver abscesses are uncommon and can be fatal if appropriate treatment is not administered. In advanced nations, bacterial or pyogenic liver abscesses (PLA) account for >80% of cases; the most common cause of such infections is hepatobiliary infection 9. The most frequently isolated pathogens in patients with PLA are reportedly *Escherichia coli*,* Klebsiella* spp., and *Enterococcus* spp. 9. According to another report, *Streptococcus* spp. are also common causative pathogens of PLA 10. Symptoms and laboratory findings associated with PLA include fever, chills, right upper abdominal pain, and high alanine aminotransferase and alkaline phosphatase concentrations; however, these findings are not definitive for PLA 9. Therefore, imaging diagnosis plays a pivotal role in establishing a definitive diagnosis of PLA. Abd‐US and Abd‐CT are the two main diagnostic tools 9. Abd‐US reportedly fails to identify PLA in approximately 15% of affected patients, most of whom have abscesses in S8, which can be difficult to examine accurately with this modality 11. Contrast‐enhanced Abd‐CT also provides useful diagnostic information for focal liver lesions; however, its accuracy depends on the size of the lesions. For example, hepatocellular carcinoma can be diagnosed with ≥90% accuracy with imaging alone; however, a radiographic diagnosis may not be possible in patients with small focal liver lesions, for whom a biopsy is therefore recommended 12. The American College of Gastroenterology Clinical Guidelines recommend the use of magnetic resonance imaging or triple‐phase CT for differential diagnosis of hepatocellular carcinoma in patients whose liver lesions are >1 cm on Abd‐US because the ability of enhanced Abd‐CT to result in an accurate diagnosis is limited by the size of the hepatic lesion 12. Moreover, characterization of focal liver lesions by Abd‐CT relies on the dynamic contrast‐enhancement characteristics of the lesions in multiple phases 13. In patients with PLA, enhanced Abd‐CT commonly shows inner lower attenuation with rim enhancement 9; however, other possible imaging findings include a solid mass‐like, cystic, or multilocular appearance 14. In the present case, the relatively small PLAs (approximately 10 mm in diameter) may have led to the diagnostic failure of enhanced Abd‐CT were it not for the relatively large lesion in S6. Thus, it is important to employ both Abd‐US and enhanced Abd‐CT to detect PLAs. In our case, the *S. constellatus* strains obtained from blood and liver tissue culture samples showed the same minimum inhibitory concentration. However, one limitation of our report was the inability to determine the genetic identity of these *S. constellatus* strains by analysis of restriction products after digestion of chromosomal deoxyribonucleic acid with restriction endonuclease.

Although the most common cause of PLA is hepatobiliary infection 9, it should be noted that embolization of septic clots resulting from intra‐abdominal infection is another important cause of PLA 10. Interestingly, colonic diverticulitis frequently causes the formation of septic clots leading to PLA, reportedly accounting for 27.5% of cases of PLA 10. Systemic hematogenous dissemination of septic clots may cause PLA, particularly in immunocompromised individuals 10. Shigefuku et al. 15 reviewed 26 cases of Japanese patients with PLA due to SMG infection. According to their report, the patients were predominantly male (85%) and their mean age was 57.5 ± 12.5 years. Multiple abscesses were observed in 10 patients (38%), and blood cultures were positive in 13 patients (57%). Pus culture was positive in all patients, revealing mixed infection with anaerobic bacteria in eight patients (31%). Although PLA due to SMG infection was observed in 10 otherwise healthy patients (38%), five of the patients (19%) with SMG‐associated PLA had cancer (one with gastric cancer and four with cholangiocarcinoma). These findings are similar to those of another study in which 14.3% of patients with SMG‐associated PLA (14.3%) had cholangiocarcinoma or gallbladder cancer 16. Thus, biliary tract cancer and subsequent hepatobiliary infection may contribute to the onset of SMG‐associated PLA. Moreover, four of the reported five patients with cholangiocarcinoma had mixed bacterial infections other than SMG, including *Enterobacter cloacae*,* Pseudomonas aeruginosa*,* Escherichia coli*,* Klebsiella pneumoniae*, and *Enterococcus faecium* 15. In contrast, all of the 10 previously healthy patients with SMG‐associated PLA were infected with SMG alone 15, suggesting that altered immune responses in patients with malignancy may partially contribute to mixed infections. Lachara et al. 17 reported a case of SMG‐associated PLA following a routine dental cleaning, suggesting that temporary bacteremia can also cause SMG‐associated PLA; this indicates the importance of hematogenous spread of SMG. In the present case, the presence of septic thrombophlebitis of the portal vein (pylephlebitis) strongly suggests that portal bacteremia led to the onset of PLA. Because it is the most common cause of pylephlebitis, diverticulitis must not be overlooked 18. A recent retrospective cohort study revealed that the incidence of PLA was 2.44‐fold higher in patients with colonic diverticular diseases 19. Moreover, the adjusted hazard ratios in patients with diverticulosis (2.26) and diverticulitis (1.98) were significantly higher than in patients without diverticular disease. However, we identified only two cases of SMG‐associated PLA secondary to diverticulitis in the PubMed database over the last four decades 20, 21; it is unclear why SMG‐associated PLA secondary to diverticulitis is rare. Further investigation is needed to clarify the mechanisms by which the SMG invades the mucous membranes of diverticulae.

In conclusion, we have herein presented a rare case of multiple SMG‐associated PLAs and thrombophlebitis of the right portal branch, probably secondary to diverticulitis of the sigmoid colon. Our case indicates the importance of the combined use of Abd‐US and enhanced Abd‐CT for both the initial diagnosis of PLA and the detection of its complications. In addition, physicians should be aware that even in healthy adults, SMG‐associated PLA can occur via hematogenous spread.

## Conflict of Interest

None declared.

## Authorship

NA: drafted the manuscript, collected the patient's data, and monitored the patient throughout the whole follow‐up period. TH: collected the patient's data. YK: collected the data. MK: helped to draft the manuscript. All authors have read and approved the final manuscript.
